# Yellow Fever Inoculation

**Published:** 1887-06

**Authors:** J. McF. Gaston

**Affiliations:** Atlanta, Ga.


### YELLOW FEVER INOCULATION.

*Editors Atlanta Medical and Surgical Journal:*

Since the inauguration of this inquiry by Dr. Friere, when I was residing in Brazil, a lively interest has been taken in every step of his investigation, and so soon as there was good and sufficient evidence to satisfy me that an important discovery had been made for the arrest of the greatest scourge of our Southern people, I set about plans for securing the consideration by the officials of our government for this matter.

After fruitless efforts to get the attention of the President and the Secretary of State for the adoption of measures to investigate the claims of yellow fever inoculation, I addressed a communication to Dr. Joseph Holt, of New Orleans, embodying my convictions of the importance attached to a solution of this question. He brought the matter before the American Health Association, and subsequently used his personal influence effectively in having a bill presented to Congress for sending a commission to Mexico and Brazil.

In the meantime, I presented a series of resolutions to the American Medical Association, authorizing a memorial in favor of the bill, and, as chairman of the committee, presented it to both houses of Congress with the signature of one other member of the committee, while the third declined to sign it as not being in accordance with his views.

While these measures were urged by the friends of inoculation, based upon the knowledge of facts to substantiate its claims to recognition, there were parties in different sections of this country whose influence was exerted to defeat the project. Strange to say, some whose interests ought to have led them to urge forward any undertaking which promised to mitigate the horrors of yellow fever were arrayed against the bill, and specious arguments were used by others based upon the manner of proceeding in accomplishing the object. If it could not be effected through a chosen channel, they determined to make war against all other means of securing the end, however justifiable this might be. Those whose private animosities and prejudices led them to oppose this prophylactic measure have trumped up ostensible objections on other grounds, which do not bear the test of a thorough scientific examination, and putting all theories of bacteriology aside, we must come down to the bald facts of experiment in judging of the protection afforded by this process of inoculation with the cultured virus of small-pox. These data have been accumulating and the impression has been seen in renewed efforts to attain the end in view. In various quarters of the globe observations have been made confirmatory of the inoculability of yellow fever and its protective effects.

This final expression of a firm conviction that preventive inoculation against yellow fever is trustworthy sustains the claim made six years ago by Dr. Domingos Friere in Rio de Janeiro. He has had to contend with difficulties which would have proved insurmountable to one who had not the courage of his convictions to urge him forward in his investigations. Like the lion-hearted Jenner, who struggled against the assaults of his own countrymen in bringing the results of vaccination against small-pox before the public, Friere has met opposition at home, as well as abroad, in prosecutiug his experiments.

But fortunate for humanity and for the cause of science, all the world has not proved deaf and blind to the demonstrations of the success of yellow fever inoculation presented by Domingos Friere in Rio de Janeiro; and I take this occasion to congratulate the people of the United States upon the appointment of Dr. Sternberg to verify the correctness of the reports presented in due form to the Brazilian government touching its protective influence upon the population.

It matters not how the result may be explained if the fact is clearly established that inoculation prevents the access of yellow fever. The question for solution is in regard to protection, and if people are rendered invulnerable by it, that settles the whole matter for us satisfactorily. The outcome of all these efforts has been a provision of $10,000 in the civil service bill for investigating the subject of yellow fever inoculation in Mexico and Brazil, and I trust we may soon realize the good results which the development of the last few years in various localities warrants us to expect.

There was a period, at the outset of this undertaking to enlist the interest of our people in this matter, when it would have suited me to co-operate in the work on a field where I was familiar with the surroundings, but the lapse of two years has so changed my professional relations as to preclude any participation in this practical inquiry touching the developments of protective yellow fever inoculation. While I cannot enter upon this noble and philanthropic undertaking, it impresses me forcibly that the duties cannot be satisfactorily performed by a single individual, and that two other medical men, with proper qualifications, should be commissioned by the President to accompany Dr. Sternberg to Mexico and Brazil. If the inquiry is to be made, let it be done properly and effectively by a commission whose decision shall inspire the confidence of our people and induce them to avail themselves of the benefits of inoculation in advance of an epidemic of yellow fever. I would not think it out of place to puton the commission one of the medical men connected with the New Orleans *Medical Journal*, which has been most active in the endeavor to thwart the enterprise, and thus afford him an opportunity to be convinced of the error of his ways, so that he may hereafter be equally zealous in advocating what may prove beneficial to the population of his city and the whole southern portion of this country.

Should none of the opponents of the measure be willing to have their eyes opened, there are various prominent members of the medical profession, who have participated in the effort to secure action by Congress, whose appointment would give general satisfaction. Among these stand conspicuous Dr. Joseph Holt, of New Orleans; Dr. Irving A. Watson, of Concord, N. H.; Dr. H. L. Davis, of Boston, and Dr. H. M. Lane, of Carthage, Mo. The last named has had practical experience of the protective influence of inoculation, and being familiar with the language and customs of the people, by long residence in Brazil, would facilitate greatly the researches in the city of Rio de Janeiro, where the most important data lie open for inspection by the commission. The names of persons inoculated, with the number of their residence and designation of the streets, have been published by authority of the Brazilian government, and the good faith of all the authorities is pledged to aid those who may be sent there in getting the facts connected with their exemption from yellow fever.

From the manifestation on the part of the Junta Hygienica (Health Association), which appeared in the public prints of Rio de Janeiro some months ago, the commission may rely upon a cordial reception by that body, and I have received from Dr. Domingos Friere on several occasions expressions of readiness on his part to render every assistance in the prosecution of this investigation, whether in the laboratory or among the population of the city, who have been subjected to his process of inoculation with cultured fluid of attenuated virus from yellow fever patients.

On the 21st of March of the current year, Dr. Friere, with Dr. Paul Gibier and Dr. C. Bebourgeon, presented to the Institution of France, in Paris, a well-elaborated statement of the results secured by various experiments upon the microbe of yellow fever and the transmissibility of the virus through it, not only to man, but the inferior animals.

The evidence of protection from yellow fever by inoculation is now so clear, and the mode in which it is accomplished is so plain, that it only requires verification to be generally adopted.

J. McF. GASTON, M. D.

*Atlanta, May 25, 1887.*

